# The Protraction and Retraction Angles of Horse Limbs: An Estimation during Trotting Using Inertial Sensors

**DOI:** 10.3390/s21113792

**Published:** 2021-05-30

**Authors:** Marie Sapone, Pauline Martin, Khalil Ben Mansour, Henry Chateau, Frédéric Marin

**Affiliations:** 1 Université de Technologie de Compiègne, UMR CNRS 7338 BioMécanique et BioIngénierie, Alliance Sorbonne Université, 60200 Compiègne, France; khalil.ben-mansour@utc.fr (K.B.M.); frederic.marin@utc.fr (F.M.); 2 Ecole Nationale Vétérinaire d’Alfort, USC INRAE-ENVA 957 BPLC, CWD-VetLab, 94700 Maisons-Alfort, France; pmartin@lim-group.com (P.M.); henry.chateau@vet-alfort.fr (H.C.); 3 LIM France, Chemin Fontaine de Fanny, 24300 Nontron, France

**Keywords:** horse, locomotion, protraction, retraction, limb angles, inertial measurement units, method comparison, biomechanics

## Abstract

The protraction and retraction angles of horse limbs are important in the analysis of horse locomotion. This study explored two methods from an IMU positioned on the canon bone of eight horses to estimate these angles. Each method was based on a hypothesis in order to define the moment corresponding with the verticality of the canon bone: (i) the canon bone is in a vertical position at 50% of the stance phase or (ii) the verticality of the canon bone corresponds with the moment when the horse’s withers reach their lowest point. The measurements were carried out on a treadmill at a trot and compared with a standard gold method based on motion capture. For the measurement of the maximum protraction and retraction angles, method (i) had average biases (0.7° and 1.7°) less than method (ii) (−1.3° and 3.7°). For the measurement of the protraction and retraction angles during the stance phase, method (i) had average biases (4.1° and −3.3°) higher to method (ii) (2.1° and −1.3°). This study investigated the pros and cons of a generic method (i) vs. a specific method (ii) to determine the protraction and retraction angles of horse limbs by a single IMU.

## 1. Introduction

The musculoskeletal system of the horse is subjected to strong mechanical stresses during sports exercise [1,2,3,4], which increase the risk of injury [5]. The integrity of the horse’s musculoskeletal system is usually assessed by a clinical examination where the veterinarian observes the horse walking and trotting “in hand” in order to detect any visible asymmetries in the movements of the head and trunk and identifies the localization of lameness [6]. The clinical examination involves particular attention to the straightness and symmetry of the horse in the gait, its attitude, the mobility of its back, its limbs and its hips [7,8]. The amplitude of movement of the limbs in the cranial and caudal phase of the stride is also a key point of observation in the analysis of the horse’s locomotion [9]. This range of motion in the cranial and caudal phase of the stride is measured by the angle of protraction and retraction of the horse’s limbs [10]. 

**Figure 1:**
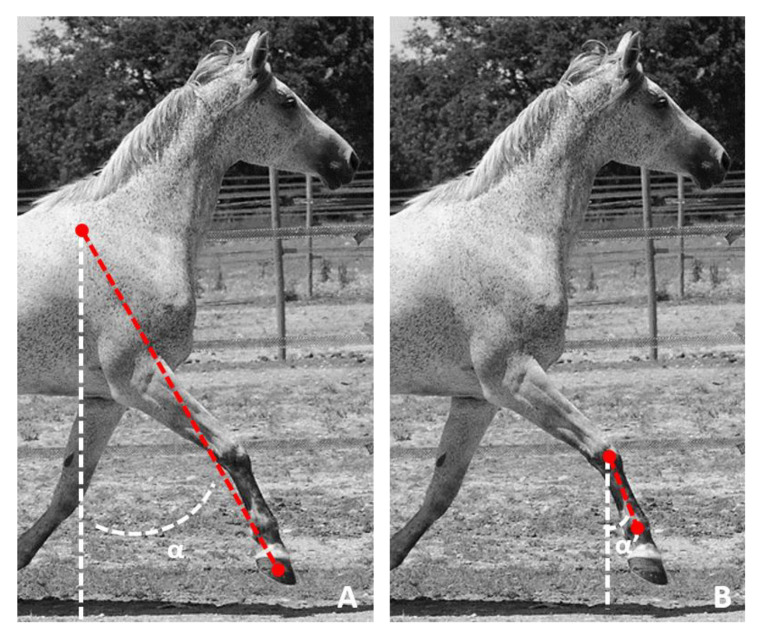
Representation of (**A**) the angle α of protraction of the whole limb measured from the orientation of the segment connecting the tuber spinae scapulae and the hoof with respect to the vertical and (**B**) of the angle α’ of protraction measured from the orientation of the canon bone (third metacarpal bone) relative to the vertical.

Commonly, limb protraction and retraction angles are defined by the angle formed by the limb’s axis relative to the vertical during the stride [11,12]. The limb’s axis is defined for the entire limb from the segment formed by the hoof and the scapula [13,14] (Figure 1A) or only for the segment of the canon bone (third metacarpal bone) [12,15] (Figure 1B). In this study we were only interested in the measurement of the protraction retraction angles measured from the segment of the canon bone (Figure 1B).

The comparison of measurements of protraction and retraction between the right and left limbs can give information on the symmetry of the locomotion of the horse and on the origin of pain [16,17,18,19,20]. Asymmetry may appear or increase due to the onset of pain or excessive fatigue in the horse [16]. For example, a sole injury at the level of the toe may be associated with an increase in the protraction of the injured limb compared with the opposite limb in order to limit the contact of the cranial part of the hoof (injured part) with the ground [17]. Conversely, an injury located at the heels is often associated with a decrease in the cranial part of the stance phase compared with the opposite limb in order to limit the pressure on the heels induced by the contact of the foot with the ground [18]. The asymmetry of protraction and retraction of the horse’s limbs can also be visible with injuries located higher in the limb. For example, a non-articular fracture of the supraglenoid tubercle often leads to a decrease of protraction of the injured limb [19]. Conversely, podotrochlear syndrome (navicular disease) is characterized by a reduction of the caudal part of the stance phase (less retraction) [20]. Consequently, the analysis of the protraction and retraction movements of the horse’s limbs and their symmetry can therefore provide useful information for the location of the injury and helps to focus the diagnosis on the area of interest. Many riders miss the appearance of asymmetry in the horse and are only alarmed when lameness is present [21]. The development of on-board measurement methods of the horse’s locomotion at work would also provide a tool for riders and trainers to detect the first signs of irregularities of the gait [22,23]. Thus, the appearance of several locomotor problems could be limited and treated more quickly by the veterinarian. This would limit the severity of the injury and optimize the recovery time. In addition, measuring the protraction and retraction angles of the horse’s limbs can also provide information on the nature of the ground [13,24] or on the comfort of the horse in relation to the saddle [25], the girth [26] or the weight of the rider [27].

In the majority of studies, the limb’s protraction and retraction measurements are performed using a motion capture system (MOCAP) [13,14,25]. Although precise, these systems involve laboratory use, which limits their applications [28]. More recently, studies have focused on the use of inertial measurement units (IMUs) to measure the protraction and retraction angles of horse limbs [12,15,22,23,24]. The use of IMUs, more versatile and pervasive [28], can then allow these measurements under the normal working conditions of the horse [22].

There are methods used to estimate the orientation of IMUs in space, in particular thanks to the Kalman filter used in many studies on human movement [29,30,31,32]. The study by [15] applied a previously developed algorithm for the analysis of human movement to the analysis of horse limb locomotion. This algorithm is based on the combined use of information from the Kalman filter applied to the signals of two synchronized IMUs [33]. Despite encouraging results, the study by [15] was limited to two horses and low speeds (a walk at 1.45 m/s and a trot at 3.18 m/s) and the application of their method therefore still requires validation. In addition, it has been shown that the performance of the Kalman filter is limited for estimating the orientation of segments that have a high velocity or impact movement [29,30,34]. Their use for the measurement of rapid locomotion of horse limbs [35] therefore requires the development of specific calculation methods [22].

More recently, other studies have used IMUs to measure the angles of protraction and retraction of the horse’s limbs [12,24]. One study used an initial phase of recording on a horse’s “halted square”, a neutral position of the horse where the limb is perpendicular to the ground, in order to register the initial orientation of the IMUs [24]. This method necessarily requires a preliminary phase to the recording of the horse’s activity, which may be restrictive depending on the conditions of use of the measurement system. To avoid this constraint, the method of calculating the protraction and retraction angles of the horse’s limbs of the second study was based on the assumption that the limb was in a vertical position (angle = 0°) at 50% of the stance phase [12]. 

The orientation of the limb can be obtained by integrating the angular velocity of the gyroscopic signal over time, paying attention to the accumulation of drift errors [36]. In order to limit the risk of drift, the integration is carried out over reduced time windows [37], corresponding in our study with the duration of the stride or stance phases (641.5 m/s and 201.5 m/s on average, respectively, at a 4 m/s trot [23]) for the measurements of the protraction/retraction movements of the limb during the stride or the stance. The gyroscopic data are recorded in the IMU’s local coordinate system. The measurement of the absolute angles of protraction and retraction of the limbs requires knowing the initial position of the limb, the neutral position, corresponding with a protraction/retraction angle of zero degrees.

The objective of this study is to evaluate and compare two integrative angular velocity methods from an IMU positioned on the middle of the dorsal face of the canon bone for the measurement of the absolute protraction and retraction angles of the horse’s limbs. Each of these methods is based on a hypothesis in order to define the moment corresponding with the verticality of the canon bone (angle = 0°). The first method is based on the hypothesis that the limb is in a vertical position at 50% of the stance phase, as suggested by [12]. The second method is based on the hypothesis that the verticality of the canon bone corresponds with the moment when the horse’s withers reach their lowest position. Indeed, observations suggest that the verticality of the canon bone is reached during the transition from damping to propulsion in the stance phase [38]. This transition also coincides with the maximum extension of the metacarpophalangeal joint [14], which is achieved when the withers are at their lowest height [19]. These two methods are compared using a reference MOCAP system to assess their accuracy.

## 2. Materials and Methods

### 2.1. Horses

Eight sound horses of trotting breeds (four geldings and four mares, height 162 ± 3 cm, age 9 ± 2 years old (mean ± sd)) from the “Centre d’Imagerie et de Recherche sur les Affections Locomotrices Equines” (CIRALE) were included in the study. All of the horses had a moderate activity. Prior to the procedure, the protocol was examined and approved by the dedicated ethics committee on animal investigations (Comité National de Reflexion Ethique sur l’Experimentation Animale, Anses/ENVA/UPEC n◦HE_2017_01).

### 2.2. Data Acquisition

The data acquisition was performed on a treadmill. Each horse trotted three times on a treadmill at a speed of 4 m/s, a common speed at which the horse is observed during clinical examinations [39,40]. They then trotted three times at a faster trotting of 6 m/s. Motion capture sessions included the tracking of 3D passive markers and IMUs [23]. Each of the eight horses was equipped with 10 reflective markers placed on an anatomical landmark [23]. In addition, two IMUs, one on the dorsal part of the canon bone and the second on the withers, were used [23]. At least 25 strides were recorded at a stabilized speed during each trial.

### 2.3. MOCAP Data Processing

**Figure 2:**
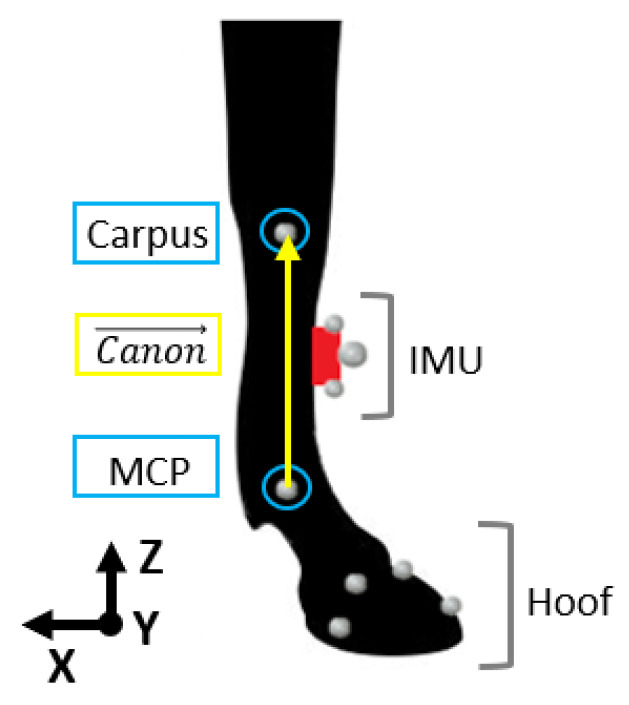
Positioning of the IMU (in red) and kinematic markers on the right front limb and orientation of the laboratory coordinate system ($\vec{x},\vec{y},\vec{z})$. The markers were positioned on specific anatomical landmarks (lateral styloid process of the radius for the carpal joint (Carpus), lateral condyle for the metacarpophalangeal joint (MCP), hoof (toe, heel, front coronary band, lateral coronary band)) and on the IMU (center, up lateral part, down lateral part). The orientation of the canon bone (in yellow) was defined by the coordinates of the Carpus and MCP markers (in blue).

The first step for the MOCAP data processing was the 3D reconstruction and labeling of the anatomical markers (Figure 2) with Nexus software (Nexus 2.8.0, Oxford Metrics Ltd., Oxford, UK).

In order to allow data windowing, the start and end events of the stance phase were then detected on the MOCAP from the hoof’s markers [23,41] (Figure 2). These events were called, respectively, FootOnMOCAP(*i*) and FootOffMOCAP(*i*) with *i* corresponding with the stride cycle and *i* equal to 1 to *n* with *n* > 25.

The calculation of the angles of the canon bone of the right forelimb was carried out from the 3D coordinate in the laboratory reference frame ($\vec{x},\vec{y},\vec{z})$ of the carpal joint marker and the metacarpophalangeal (MCP) joint marker (Figure 2).

$\vec{Carpus}\left(j\right)$ and $\vec{MCP}\left(j\right)$ were the 3D coordinates of the carpal joint and the MCP at the *j*-th frame of each stride *i*. The orientation of the canon bone was then defined by Equation (1):

(1) $$\vec{Canon}\left(j\right)=\frac{\vec{Carpus}\left(j\right)-\vec{MCP}\left(j\right)}{\vec{Carpus}\left(j\right)-\vec{MCP}\left(j\right)}.$$

The angle of the canon bone for measuring the protraction and retraction movements was defined as the orientation of the canon bone in the sagittal plane [12,13,15] and called AngleCanonMOCAP(*j*). The AngleCanonMOCAP(*j*) was calculated from the coordinates of the canon vector at each frame (*j*) of each stride (*i*) measured (Equation (2)).

(2) $$\mathrm{AngleCanonMOCAP}\left(i\right)=\;\left\{\mathrm{atan}2\left(\vec{Canon}\left(j\right).\vec{x},\vec{\mathrm{Canon}}\left(j\right).\vec{z}\right)|\;j\in \left[\mathrm{FootOnMOCAP}\left(i\right)\;\mathrm{FootOnMOCAP}\left(i+1\right)-1\;\right]\right\}.$$

**Figure 3:**
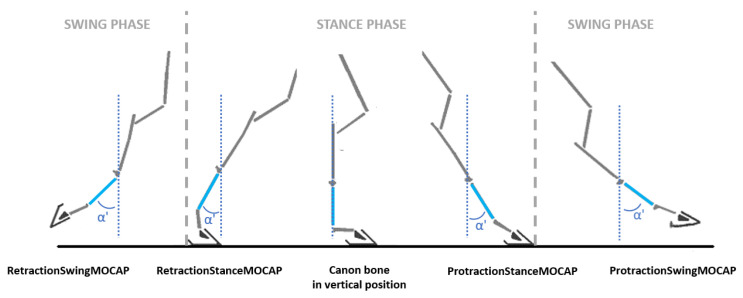
Graphic representation of the moments of the stride at which the angular values α’ of protraction and retraction were measured. (Adapted from the illustration by [42]).

For each of the recorded strides, the calculation of the protraction angle and the retraction angle was then deduced from the canon angle in two ways:

(1) During the stance phase when the limb was in protraction at the start of the stance phase (initial contact of the hoof flat on the ground) and when the limb was in retraction at the end of the stance phase, at the moment preceding the breakover of the hoof. These events were called, respectively, ProtractionStanceMOCAP(*i*) and RetractionStanceMOCAP(*i*) with *i* corresponding with the stride cycle ranging from 1 to *n* (*n* > 25) for each recorded trial (Figure 3).
(2) During the swing phase, when the limb was in maximum extension, which corresponded with the maximum protraction and when the limb was in the maximum retraction. These events were called, respectively, ProtractionSwingMOCAP(*i*) and RetractionSwingMOCAP(*i*) with *i* corresponding with the stride cycle ranging from 1 to *n* (*n* > 25) for each recorded trial (Figure 3).

**Figure 4:**
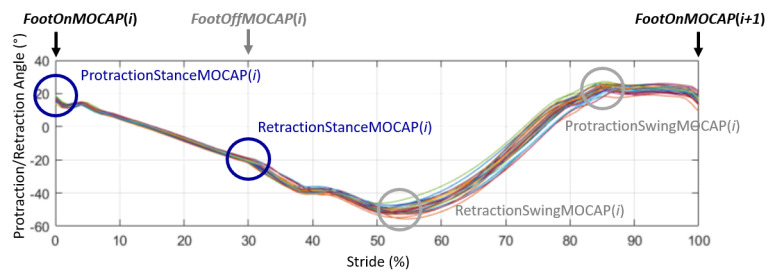
Representation of the canon bone orientation obtained from the MOCAP data for the 26 normalized strides of a trial of one horse trotting at a 4 m/s speed. The maximum and minimum points at each stride, corresponding, respectively, with the ProtractionSwingMOCAP and RetractionSwingMOCAP of the right forelimb, are circled in grey on the Figure. The angles of protraction and retraction at the stance correspond, respectively, with the angles of the canon bone at Foot On and Foot Off, represented by blue circles in the Figure.

The values of protraction and retraction of the limb during the stance phase corresponded, respectively, with the values of the orientation of the canon bone at Foot On and to the values of the orientation of the canon bone at Foot Off for each stride (*i*) (Equations (3) and (4)) (Figure 4).

(3) ProtractionStanceMOCAP(i)=AngleCanonMOCAP(i){1}

(4) RetractionStanceMOCAP(i)=AngleCanonMOCAP(i){FootOffMOCAP(i)−FootOnMOCAP(i)+1}.

The maximum value on the orientation of the canon bone during the swing phase was identified on each stride corresponding with ProtractionSwingMOCAP(*i*) (Equation (5)) (Figure 4). The minimum value, in an absolute value, corresponding with RetractionSwingMOCAP(*i*) (Equation (6)) was also identified on each stride (Figure 4).

(5) ProtractionSwingMOCAP(i)=max(AngleCanonMOCAP(i))

(6) RetractionSwingMOCAP(i)=abs(min(AngleCanonMOCAP(i))).

### 2.4. Data Processing of the IMUs 

The calculation of the canon angle by the IMU was performed by the integration of the gyroscope data, which required an integrative constant to be determined. The first step in processing the IMU data was also to define a reference point where the canon bone was at a vertical position corresponding with a limb protraction/retraction angle of 0°. For this, two methods were used and compared:

(i) The canon bone was considered in a vertical position at 50% of the stance phase [12] (“50%_Stance” method).
(ii) The canon bone was considered in a vertical position at the moment when the withers reached their lowest point (“minWithers” method).

In the case of (i), the index corresponding with 50% of the stance phase was calculated from the FootOnMOCAP(*i*) and FootOffMOCAP(*i*) data previously defined on the MOCAP data for each stride *i* ranging from 1 to *n* (*n* > 25). This gave the index 50% _Stance(*i*) for each stride recorded (Equation (7)).

*50%_Stance(i) = (MOCAPFootOff(i) − MOCAPFootOn(i))/2*. (7)

**Figure 5:**
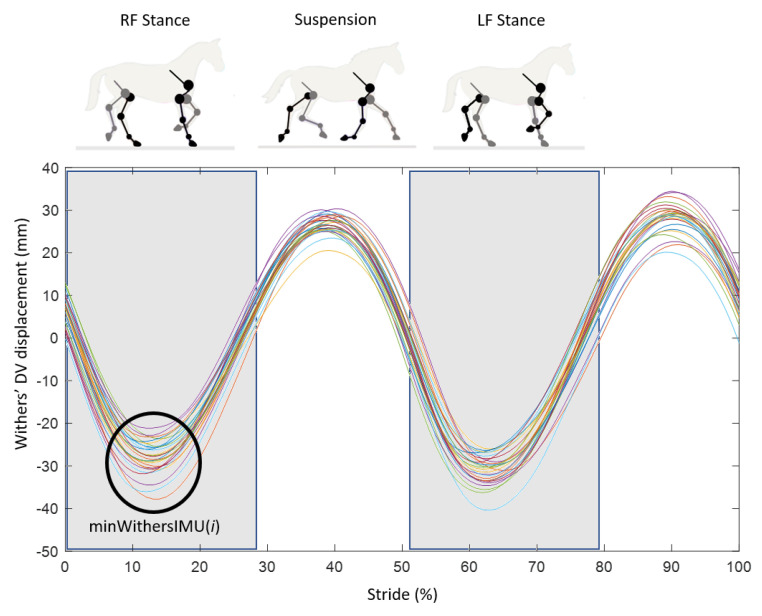
Example of dorso-ventral (DV) displacement curves obtained from the IMU of the withers at a 4 m/s trot (26 standardized strides shown) for one of the eight horses that participated in the experimental protocol. For each stride, the first minimum point is obtained during the first oscillation corresponding with the stance of the right front limb (RF) and named minWithersIMU(*i*). It is followed by a suspension phase before reaching a second minimum point during the second oscillation corresponding with the stance of the left front limb (LF).

In the case of (ii), the vertical displacement of the withers was calculated from the MOCAP data and the IMU data. For the MOCAP data, the vertical position of the withers corresponded with the coordinates of the marker of the withers on the Z axis (the vertical axis in the laboratory reference system). From the IMU, the vertical displacement of the withers was obtained by a double trapezoidal cumulative integration of the acceleration on the Z axis of the IMU [43,44] with the application of a Butterworth high pass filter (1 Hz) to remove the drifts generated by the double integration. The dorso-ventral displacement curves of the withers at the trot obtained from the MOCAP and IMU data formed two oscillations repeated at each stride corresponding with the stance of each of the horse’s forelimbs [44,45] (Figure 5). The detection of the minimum position of the withers was performed for the oscillation corresponding with the stance of the right forelimb (Figure 5) on the displacement curve of the withers obtained from the MOCAP data and the displacement curve of the withers obtained from the IMU data. The detection of the minimum position of the withers was made only on the oscillation corresponding with the stance of the right forelimb because it was the limb equipped with the IMU. The correlation coefficient between the indices corresponding with the minimum position of the withers obtained from the MOCAP data and those obtained from the IMU data showed a perfect correlation (R = 1 with α = 0.01), leading us to choose to use only the indices obtained from IMU data for the continuation of the data processing. Thus, the frame minWithersIMU(*i*) was obtained for each stride *i* ranging from 1 to *n* (*n* > 25) (Figure 5).

Once the 50%Stance(*i*) and minWithersIMU(*i*) indices were obtained for each stride *i* recorded, the calculation of the orientation of the canon bone from the data of the IMU positioned on the canon bone could be achieved in two steps.

**Figure 6:**
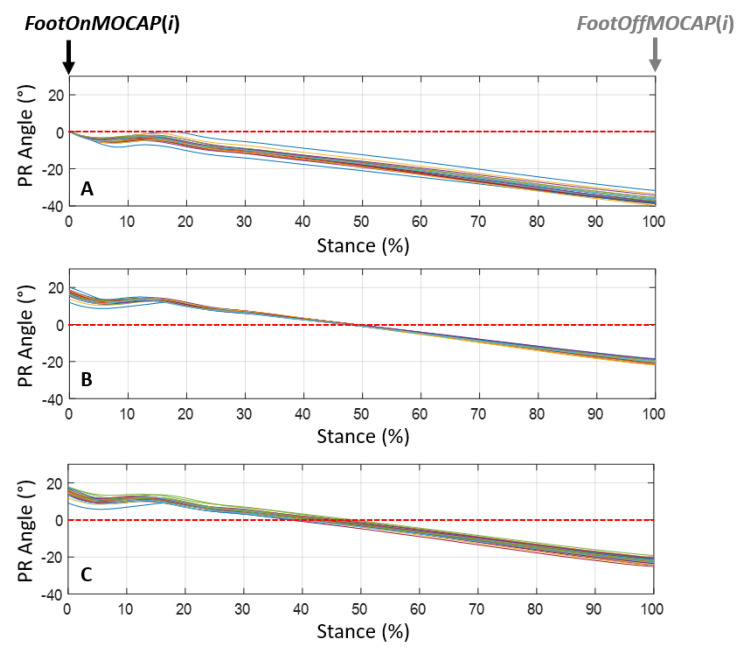
Protraction/retraction angles (PR) normalized by the stance phase obtained by calculating the cumulative trapezoidal integral without resetting, named AngleCanonIMU_*temps*(*i*) (**A**), with resetting at the index 50% Stance(*i*), named AngleCanonIMU_*50%S*(*i*) (**B**) and with resetting to the index minWithersIMU(*i*), named AngleCanonIMU_*minW*(*i*) (**C**) of the gyroscope signal on the Y axis on the 26 strides recorded during a trial at a 4 m/s trot of one horse (*i* ranging from 1 to 26).

First, the signal from the gyroscope on the Y axis ($\omega IMU_{y}$) at each frame was integrated to determine the temporary values of the canon angle based on the IMU data for each stride *i* (AngleCanonIMU_*temp(i))* as follows (Equation (8)) (Figure 6A):

(8) $${\mathrm{AngleCanonIMU}}_{temp}\left(i\right)=\left\{\frac{1}{f}\int_{\mathrm{FootOnIMU}\left(i\right)\;}^{j}\omega IMU_{y}\left(\lambda \right)d\lambda \;|\;j\in \left[\mathrm{FootOnIMU}\left(i\right)\;\mathrm{FootOnIMU}\left(i+1\right)-1\;\right]\right\}.$$

The numerical calculation of the integration was performed by a trapezoidal method.

Let us note that AngleCanonIMU_temp(i){k} was the k-th element of AngleCanonIMU_temp(i). At this stage, let us remark that at each stride *i*, we had AngleCanonIMU_*temp(i)*{1} = 0° (Figure 6A).

We could then add the integrative constant to deduce the canon angle according to option (i) and (ii), respectively, named AngleCanonIMU_*50%S* (Equation (9)) (Figure 6B) and AngleCanonIMU_*minW* (Equation (10)) (Figure 6C) and defined as follows:

(9) $$\begin{array}{l} {\mathrm{AngleCanonIMU}}_{50}\%S\left(i\right)=\\ \mathrm{AngleCanonIMU}\_temp\left(i\right)-\mathrm{AngleCanonIMU}\_temp\left(i\right)\left\{50\%\mathrm{Stance}\left(i\right)-\mathrm{FootOnIMU}\left(i\right)+1\right\} \end{array}$$

(10) $$\begin{array}{l} {\mathrm{AngleCanonIMU}}_{minW\left(i\right)}=\\ \mathrm{AngleCanonIMU}\_temp\left(i\right)-\mathrm{AngleCanonIMU}\_temp\left(i\right)\left\{\mathrm{minWithersIMU}\left(i\right)-\mathrm{FootOnIMU}\left(i\right)+1\right\} \end{array}$$

We could then compute the protraction and retraction angle during the stance phase according to option (i) and (ii) as follows (Equations (11)–(14)):

(11) ProtractionStanceIMU_50%S (i)=AngleCanonIMU_50%S(i){1}

(12) ProtractionStanceIMU_minW (i)=AngleCanonIMU_minW(i){1}

and,

(13) RetractionStanceIMU_50%S(i)=AngleCanonIMU_50%S(i){FootOffIMU(i)−FootOnIMU(i)+1}

(14) RetractionStanceIMU_minW(i)=AngleCanonIMU_minW(i){FootOffIMU(i)−FootOnIMU(i)+1}.

Finally, the maximal value of protraction and retraction during the stride according to option (i) and (ii) could be deduced as follows (Equations (15) to (18)):

(15) ProtractionSwingIMU_50%S(i)=max(AngleCanonIMU_50%S(i))

(16) ProtractionSwingIMU_minW(i)=max(AngleCanonIMU_minW(i))

and,

(17) RetractionSwingIMU_50%S(i)=abs(min(AngleCanonIMU_50%S(i)))

(18) RetractionSwingIMU_minW(i)=abs(min(AngleCanonIMU_minW(i))).

### 2.5. Statistical Analysis

The results of each method with the IMU data were then compared with the MOCAP data using Bland–Altman plots [46]. For each method, the accuracy was defined by the mean difference (bias) between the values of the developed method and the reference values obtained from the MOCAP data and the precision such as the standard deviation of differences (SD). The limits of agreement corresponded with the confidence interval where 95% of the differences were calculated [46]. The bias and the SD were used to estimate this interval (Equations (19) and (20)). The results were expressed as the difference in degrees between the angles measured using the method applied to the IMU and those determined with the MOCAP.

AgreementLimitHigh_X = Bias + 1.96 × SD(19)

AgreementLimitLow_X = Bias + 1.96 × SD.(20)

## 3. Results

### 3.1. Measurement of the Protraction Angles

**Figure 7:**
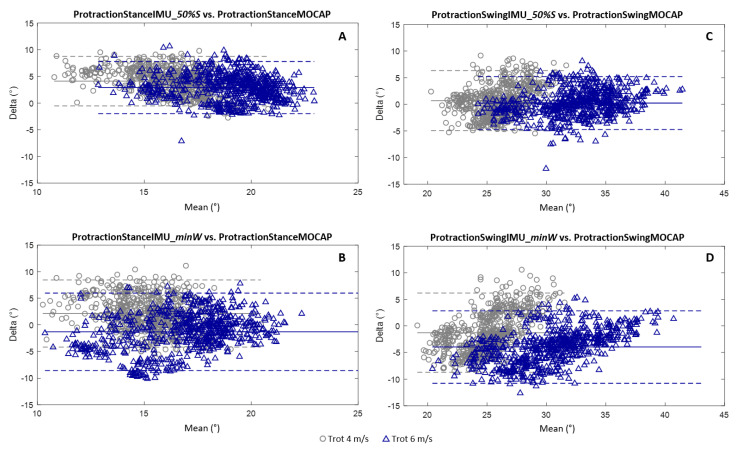
Bland–Altman graphs for the comparison of the protraction angles at a stance obtained using the indices 50%Stance (**A**) and minWithers (**B**) and of the angles of maximal protraction obtained using the indices 50%Stance (**C**) and minWithers (**D**) with the protraction angles, respectively, at a stance and at a swing obtained from the MOCAP data. The results for the 4 m/s trot are shown in gray (o) and the results for the 6 m/s trot are shown in blue (Δ). The accuracy (bias between the developed method and the MOCAP) and the limits of the confidence interval (95% of values) are shown for each graph.

For the calculation of the protraction angles at a stance, the 50%Stance method (Figure 7A) had an average bias greater than the minWithers method (Figure 7B) for the two trotting speeds tested (4 m/s and 6 m/s). The values of these biases were 4.1° at a 4 m/s trot and 2.9° at a 6 m/s trot for the 50%Stance method vs. 2.1° at a 4 m/s trot and −1.3° at a 6 m/s trot for the minWithers method. Conversely, the confidence interval was more restricted for the 50%Stance method for the two speeds tested (−0.5°and 8.7° at a 4 m/s trot; −2.0° and 7.8° at a 6 m/s trot) than for the minWithers method (−4.2° and 8.4° at a 4 m/s trot; −8.6° and 6.0° at a 6 m/s trot). In addition, the 50%Stance method tended to overestimate the protraction values at a stance for the two trotting speeds tested.

For the calculation of the maximum protraction angles at a swing, the 50%Stance method (Figure 7C) presented an average bias less than the minWithers method (Figure 7D) for the two tested trotting speeds (4 m/s and 6 m/s). The values of these biases were 0.7° at a 4 m/s trot and 0.2° at a 6 m/s trot for the 50%Stance method vs. −1.3° at a 4 m/s trot and −4.0° at a 6 m/s trot for the minWithers method. The confidence interval was also more restricted for the 50%Stance method for the two speeds tested (−4.9° and 6.3° at a 4 m/s trot; −4.7° and 5.2° at a 6 m/s trot) than for the minWithers method (−8.7° and 6.2° at a 4 m/s trot; −10.8° and 2.8° at a 6 m/s trot). In addition, the increase in speed tended to increase the measurement bias with the minWithers method for the calculation of the maximum protraction angles at a swing.

### 3.2. Measurement of the Retraction Angles

**Figure 8:**
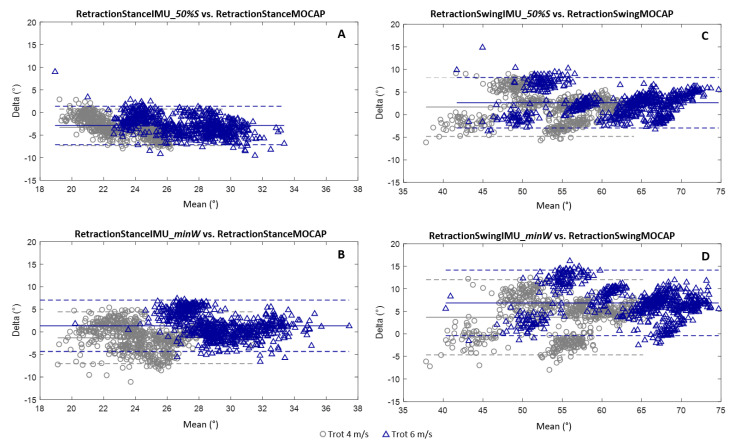
Bland–Altman graphs for the comparison of the retraction angles at a stance obtained using the indices 50%Stance (**A**) and minWithers (**B**) and of the angles of maximal retraction obtained using the indices 50%Stance (**C**) and minWithers (**D**) with the retraction angles, respectively, at a stance and at a swing obtained from the MOCAP data. The results for the 4 m/s trot are shown in gray (o) and the results for the 6 m/s trot are shown in blue (Δ). The accuracy (bias between the developed method and the MOCAP) and the limits of the confidence interval (95% of values) are shown for each graph.

For the calculation of the retraction angles at a stance, the 50%Stance method (Figure 8A) had an average bias greater than the minWithers method (Figure 8B) for the two trotting speeds tested (4 m/s and 6 m/s). The values of these biases were −3.3° at a 4 m/s trot and −2.9° at a 6 m/s trot for the 50%Stance method vs. −1.3° at a 4 m/s trot and 1.3° at a 6 m/s trot for the minWithers method. Conversely, the confidence interval was more restricted for the 50%Stance method for the two speeds tested (−7.3° and 0.7° at a 4 m/s trot; −7.1° and 1.4° at a 6 m/s trot) than for the minWithers method (−7.0° and 4.4° at a 4 m/s trot; −4.3° and 7.0° at a 6 m/s trot). It was also observed that the 50%Stance method tended to underestimate the stance retraction values for the two trotting speeds tested.

For the calculation of the maximum retraction angles at a swing, the 50%Stance method (Figure 8C) had an average bias less than the minWithers method (Figure 8D) for the two trotting speeds tested (4 m/s and 6 m/s). The values of these biases were 1.7° at a 4 m/s trot and 2.6° at a 6 m/s trot for the 50%Stance method vs. 3.7° at a 4 m/s trot and 6.8° at a 6 m/s trot for the minWithers method. The confidence interval was also more restricted for the 50%Stance method for the two speeds tested (−4.8° and 8.2° at a 4 m/s trot; −3.0° and 8.2° at a 6 m/s trot) than for the minWithers method (−4.7° and 12.0° at a 4 m/s trot; −0.4° and 14.1° at a 6 m/s trot). We also observed an increase in the average bias for the calculation of the maximum retraction angles at a swing with increasing speed for both methods. In addition, the increase in speed tended to increase the measurement bias with the minWithers method for the calculation of the maximum retraction angles at a swing. A wider confidence interval was also observed on the Bland–Altman graphs for the measurement of the retraction angle during the swing phase.

## 4. Discussion

The protraction and retraction angles of the limbs are important parameters in the analysis of the locomotion of a horse. Asymmetry in the limb protraction and retraction angles is generally associated with lameness [17,18,19,20]. An analysis of these movements can therefore provide useful information for a diagnosis. 

Classically, studies use MOCAP to measure the protraction and retraction angles of the horse’s limbs [13,14,25]. The precision of the MOCAP makes it a benchmark for studying the biomechanics of horse limbs but its use in the laboratory limits its field of application. Recently, other studies proposed an investigation into the protraction and retraction angles of horse limbs by the use of IMUs [12,15,24]. Inertial sensors, i.e., IMUs, are more versatile [28] and they can allow recordings in real equestrian conditions. However, they require the use of specific methods to estimate the spatial orientation [28]. In this study, using a strapdown inertial navigation system approach [47], the orientation of the IMU positioned on the horse’s canon bone was computed by the integration of the gyrometer data. The gyroscopic signal is known to drift over time [30,48]. In order to limit the drift, these methods were applied over small time windows [37] corresponding in our study with the duration of the stride or stance phases (641.5 m/s and 201.5 m/s on average, respectively, at a 4 m/s trot [23]). The results obtained for the measurement of the maximum protraction and retraction angles were of the same order of magnitude as those measured by [12]. The increase in the range of motion of the limbs with an increasing speed was also in agreement with the study of [40]. This increase in the range of motion of the limbs, linked to the increase in speed [40], could lead to more skin movement particularly during the swing phase [49]. Indeed, a wider confidence interval was observed on the Bland–Altman graphs for the measurement of the retraction angle during the swing phase. Nevertheless, the Bland–Altman graphs did not show a link between the measurement bias and the range of motion of the limb.

As the method used here was based on the integration of the gyrometer data, it required the definition of an integrative constant. This involved identifying the time when the horse’s canon bone was vertical, i.e., corresponding with a protraction/retraction angle of zero degrees. For this, two methods were tested. The first method, named the “50%Stance” method, was based on the hypothesis developed by Bosh et al. [12] assuming that the horse’s canon bone reaches its verticality at 50% of the stance phase. This choice was made by the authors because it defined a reliable and easily identifiable position in the stride and it was still quite close to the vertical orientation of the canon bone [12]. 

The second method, named the “minWithers” method, was based on the hypothesis that the verticality of the canon bone is linked with the minimum altitude of the withers. Observations indeed suggest that the verticality of the canon bone is reached during the transition from damping to propulsion at the stance phase [38]. In addition, this transition also coincides with the maximum extension of the metacarpophalangeal joint [14], which is reached when the withers are at their lowest height [19]. It has also been shown that the asymmetry of the vertical movement of the withers is associated with a pattern of asymmetry in the protraction and retraction of the limbs [50]. At this stage, both methods seemed to be consistent. However, the 50%Stance method could be seen as an average behavior and the minWithers method could be seen as a biomechanical deduction of the horse’s locomotion. The results of both methods were contrasted. The maximum protraction and retraction angles during the swing phase of the limb had a lower bias with the 50%Stance method than the minWithers method. Meanwhile, the protraction and retraction values of the limb at the beginning and end of the stance phase showed lower biases with the minWithers method. In addition to the better results obtained on the estimation of the values during the stance phase, one advantage of the minWithers method was its specificity to each stride, allowing the adjustment of the point in time when the canon bone was considered to be in a vertical position. However, the minWithers method required the use of an additional IMU to those of the limbs, fixed at the withers. The use of this additional IMU could then be a limit to the system. Indeed, this additional data had its own noise to signal ratio and uncertainty due to the attachment system of the withers in particular, which could contribute to increasing the measurement variability. Considering this observation, the use of the 50%Stance index for the measurements of the maximum protraction and retraction angles of the limb during the swing phase had advantages because it did not require the addition of an IMU on the withers. Our result suggested that it also had disadvantages, in particular by the loss of precision during the measurements of the protraction/retraction angles at a stance, which could be a limit to the system. Indeed, this point could be critical in the case of a lame horse because the asymmetry of the gait may be marked by a modification of the cranial [17,18,20] or caudal part of the stance phase, which is probably the most important to consider [18]. The use of a fixed common percentage of the stance phase by the 50%Stance method to determine the verticality of the canon over-evaluated the symmetrical behavior of the horse’s locomotion; a hypothesis that is difficult to support clinically [51,52]. 

On a lame horse, the movement of the withers is also asymmetric in amplitude [51,52] but we currently lack information on the temporal characteristics of the movements of the withers of a lame horse. The use of the minWithers index to define the verticality of the canon bone and to deduce the absolute value of the protraction and retraction angles of the limbs of the lame horse therefore requires further investigation.

In order to determine the most efficient method for the integration into a tool for measuring the locomotion of horse limbs, these methods would therefore need to be tested in the horse’s normal working condition, on different ground, gaits and speeds as well as on lame horses.

This study makes it possible to consider methods of analyzing horse locomotion integrated into an on-board system to enable veterinarians to use it not only in the laboratory but also in real conditions. This would thus provide quantified data in addition to the observations made by the clinician for the monitoring of the locomotion of the horse.

## 5. Conclusions

Methods were developed for the evaluation of the protraction and retraction angles of the limbs of a horse based on two different hypotheses. The first used a fixed index 50%Stance to define the moment corresponding with the verticality of the canon bone (0° angle) while the second depended on the position of the withers at their lowest point, called minWithers, at each stride. The use of the 50%Stance method showed lower biases for the measurements of the maximum protraction and retraction angles of the limb during the swing phase. For the measurement of the protraction/retraction angles at the beginning and end of the stance phase, the use of the minWithers method showed lower biases. Contrary to the 50%Stance method, the use of the minWithers method required the addition of an IMU on the horse’s withers. Even if the 50%Stance method was easier to set up with a wearable device, its application in real working conditions and in lame horses remains questionable.

Our study showed the feasibility of estimating the protraction and retraction angles of a horse’s limbs with a single IMU fixed on the canon. A further application could be to measure the symmetry of these movements and their evolution over time and conditions. Thus, the use of an on-board system for measuring the locomotion of the horse’s limbs could make it possible to detect an asymmetry of the protraction/retraction angles that may be associated with pain or excessive fatigue in the horse and also help to locate the injured area. Thus, the horse could benefit from an adapted activity and a rapid treatment by the veterinarian to limit the appearance of injuries, their severity and optimize the recovery time. This would be beneficial for the health of the horse but also for the owner by limiting the cost of associated care.
